# Mice Carrying Unmutated Human SIGLEC10 Gene Cluster Transgene Develop Both Amyloid Plaques and Tau Neurofilament Tangles: A Model for Late Onset Alzheimer’s Disease

**DOI:** 10.1002/alz70859_101773

**Published:** 2025-12-25

**Authors:** Peng Wang, Ido Weiss, Pan Zheng, Yang Liu

**Affiliations:** ^1^ OncoC4, Inc., Rockville, MD USA

## Abstract

**Background:**

Genome‐wide association studies (GWASs) suggested associations between late‐onset Alzheimer’s Disease (AD) and polymorphisms in several Siglec genes in chromosome 19q13(1,2). In the brain, SIGLEC10 is exclusively and abundantly expressed in microglia. Spatial mRNA analysis from AD brain revealed increased Siglec10 mRNA proximal to Aβ plaques over distant brain tissues. Moreover, SIGLEC10 is progressively elevated in AD tauopathy. However, no data showed causative relationship between SIGLEC10 expression and AD pathogenesis.

**Method:**

To examine the potential role for SIGLEC10 in AD pathogenesis, we created a transgenic mice line using human genomic fragment encompassing several SIGLECS including SIGLEC10. The transgene is crossed into mice with targeted deletion of *SiglecG*, the putative orthologue of human SIGLEC10. The transgenic mice and age‐match syngeneic WT control mice were assessed for accumulation of Ab amyloid and pTau neurofilament tangles (NFT) by immunohistochemistry and the contribution of SIGLEC10 is confirmed by using an anti‐SIGLEC10 mAb.

**Result:**

Flow cytometry of single cell suspension of the brain tissue confirmed the microglia exclusive expression of the human SIGLEC10 among brain cells. Importantly, 9‐13 months old SIGLEC10 transgenic mice exhibit marked increase of both NFT (immunostained by anti‐phospho‐Tau (Ser202, Thr205) antibody AT8) and Aβ plaques (immunostained by anti‐beta amyloid Ab1‐42 antibody H31L21). To confirm contribution of SIGLEC10 to accumulation both Tau and Aβ pathology, mice were treated with either vehicle or anti‐SIGLEC10 antibody for 1 month and compared the accumulation of these protein aggregates in the brain. The results show significant reduction of the pTau aggregates and Aβ plaques by anti‐SIGLEC10 mAb.

**Conclusion:**

Mice with transgenic expression of unmutated human SIGLEC10 gene cluster in microglia presented with marked increase of both NTF and Aβ plaques at 9 to 13 months. Short‐term 4‐week anti‐Siglec10 treatment significantly reduced both NTF and Aβ plaques in transgenic mice. These data demonstrate a causal relationship between SIGLEC10 and pathogenesis of late onset AD which is not associated with pathogenic mutations in early onset AD, such as APOE4, APP, PS1, PS2 or MAPT. To our knowledge, this is the first mouse model in which a single un‐mutated human gene cluster exacerbate both pTau aggregates and Aβ plaques, two critical AD hallmarks.

